# Review on Progress of Lamellar Orientation Control in Directionally Solidified TiAl Alloys

**DOI:** 10.3390/ma16134829

**Published:** 2023-07-05

**Authors:** Han Liu, Xianfei Ding, Xiao Zong, Hong Huang, Hai Nan, Yongfeng Liang, Junpin Lin

**Affiliations:** 1 Cast Titanium Alloy R&D Center, Beijing Institute of Aeronautical Materials, Beijing 100095, China; 2 Beijing Engineering Research Center of Advanced Titanium Alloy Precision Forming Technology, Beijing 100095, China; 3 Beijing Institute of Aeronautical Materials Co., Ltd., Beijing 100094, China; 4 State Key Laboratory for Advanced Metals and Materials, University of Science and Technology Beijing, Beijing 100083, China

**Keywords:** TiAl alloys, directional solidification, lamellar orientation, seed crystal, solidification parameters

## Abstract

TiAl alloys have excellent high-temperature performance and are potentially used in the aerospace industry. By controlling the lamellar orientation through directional solidification (DS) technology, the plasticity and strength of TiAl alloy at room temperature and high temperatures can be effectively improved. However, various difficulties lie in ensuring the lamellar orientation is parallel to the growth direction. This paper reviews two fundamental thoughts for lamellar orientation control: using seed crystals and controlling the solidification path. Multiple specific methods and their progress are introduced, including α seed crystal method, the self-seeding method, the double DS self-seeding method, the quasi-seeding method, the pure metal seeding method, and controlling solidification parameters. The advantages and disadvantages of different methods are analyzed. This paper also introduces novel ways of controlling the lamellar orientation and discusses future development.

## 1. Introduction

The development of the aviation and aerospace industries raises higher requirements for high-temperature structural materials. Due to their low density, high specific strength, and notable high-temperature performance, TiAl alloys are gradually becoming a new generation of high-temperature structural materials, possibly replacing some Ni-based superalloys in the near future [1,2,3]. However, traditional TiAl alloys possess low plasticity and poor creep properties at room temperature or high temperatures, which limits their further application [4,5,6].

Directional solidification (DS) technology is beneficial to control the consistency of lamellar orientation and enhancing the high-temperature plasticity and creep properties of TiAl alloys [7]. Previous research has shown that directionally solidified TiAl alloys with aligned lamellae have diverse mechanical properties in different directions [8,9]. When the lamellar orientation is perpendicular to the load direction, the plasticity is relatively poor. When the lamellar orientation is parallel to the load direction, the tensile strength and elongation at room temperature are balanced and relatively high [10]. For example, Chen et al. reported that Ti-45Al-8Nb PST single crystal alloy with aligned parallel lamellae had a yield strength of 708 MPa and an average tensile ductility of 6.9% at room temperature. Moreover, at high temperatures, it still maintained the anisotropy of mechanical properties [11]. For aero-engines, the stress borne by their turbine blades is mainly unidirectional. The application of directionally solidified TiAl alloys can effectively reduce the structural weight and improve the thrust-to-weight ratio of the engine.

In previous studies, researchers have proposed two fundamental theories for controlling lamellar orientation. One is using a certain seed alloy, of which the primary phase is α or β and the solidification phase is α. It has a stable or nearly stable lamellar structure [12]. The seed crystal is placed with lamellae parallel to the growth direction to align the lamellar orientation of the master alloy. The other theory is controlling the solidification path, ensuring the leading phase is β. According to Burgers’ relationship {110}_β_//(0001)_α_ and <111>_β_‖<$11\bar{2}0$>_α_ and Blackburn’s relationship (0001)_α_‖{111}_γ_ and <$11\bar{2}0$>_α_‖<110>_γ_, since the preferred orientation of β phase is <100>, the final lamellar orientation is 0° or 45° to the growth direction [13].

Based on these two fundamental theories, the methods of controlling lamellar orientation can be developed from the perspectives of deciding seed alloy composition and adjusting solidification parameters. The purpose of this paper is to summarize the theories of lamellar orientation control, describe the application scope and mechanism of various methods, and look forward to novel methods for lamellar orientation control.

## 2. Difficulties and Critical Problems in Lamellar Orientation Control

At present, the methods of lamellar orientation control are mostly applied in the laboratory; there are few reports on the industrialized production of directionally solidified TiAl alloy bars or components. The solidification modes of multi-component TiAl alloys are not clear yet, bringing difficulties in obtaining single crystal or columnar crystals with consistent lamellar orientation. Furthermore, the application range of traditional α phase seeds remains relatively limited; the quasi-seed method [14,15] and the self-seeding method [16,17,18] require specific master alloy compositions. 

**Figure 1:**
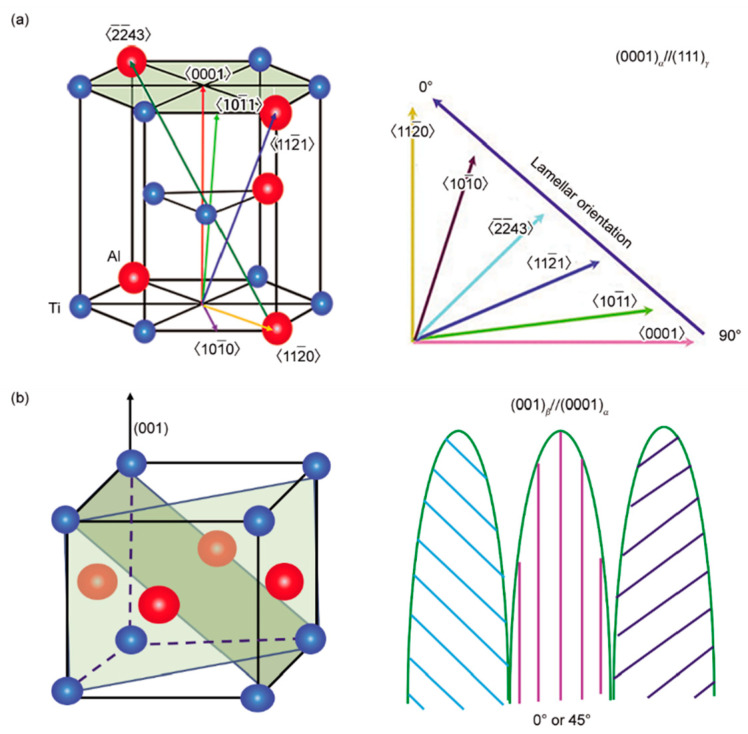
Schematics of the relationship between the lamellar orientation and the growth direction of the primary phases: (**a**) crystal orientation and lamellar orientation of α phase; and (**b**) crystal orientation and lamellar orientation of β phase [19].

A direct relationship exists between lamellar orientation and the growth direction of the primary phase, as shown in Figure 1 [19]. Nevertheless, factors affecting solidification paths are complicated and diverse. On the one hand, alloy composition directly affects the solidification process. Ding et al. [20] proposed that under the same solidification parameters, as the composition varies, there are four different solidification modes for different Nb-TiAl alloys, namely, single β, hypo-peritectic, hyper-peritectic, and single α. On the flip side, the solidification parameters play a critical role in determining the solidification path of a certain TiAl alloy. Growth rate and temperature gradient show great influence on the solidification paths. In most cases, certain solidification modes need to be determined by experiments.

Yamaguchi et al. [21,22,23,24,25,26,27] have developed a series of seed alloys suitable for DS by the optical floating zone method, such as Ti-43Al-3Si, Ti-46Al-1.5Mo-(1.0,1.2,1.5) Si, Ti-46Al-1.5Mo-0.2C, etc. Kim et al. have developed Ti-46Al-3Nb-0.6Si-0.2C [28]. Although Ti-43Al-3Si has been applied to some extent, the composition is generally different from master alloys, resulting in an uneven composition and a relatively long transition zone [12] after DS, which reduces the mechanical properties of the materials. In previous studies, the samples obtained by DS through the floating zone method had better structure and mechanical performance, but their size remained far from practical application. In order to realize the industrial application of directionally solidified alloys, a sample should possess a columnar or single crystal structure, and the lamellar orientation should be parallel to the growth direction. However, due to heat flow deflection, mold deformation, and the limitation of the continuity of grain growth, it is difficult to obtain lamellar orientation consistency in large specimens.

## 3. Seed Crystal Method and Principles

The seed crystal methods are efficient and mature in preparing Ni-based superalloy single crystal turbine blades. For TiAl alloys, studies found that the fundamental principle of lamellar orientation control was that the properly aligned lamellar structure was generated by α → α/γ → α_2_/γ or α → α_2_ → α_2_/γ phase transition. The seed technique uses TiAl alloy with parallel lamellae as seed crystal for directional solidification; the leading α phase grows along its non-preferred <$11\bar{2}0$> orientation. Consequently, a TiAl alloy with a full lamellar structure completely parallel to the growth direction is prepared. Moreover, the leading α phase can be obtained by peritectic solidification or generated under certain conditions, which gives rise to other seed methods besides the traditional α seed crystal method, including the self-seeding method, double DS self-seeding method, quasi-seeding method, and pure metal seeding method.

### 3.1. α Seed Crystal Method

Yamaguchi et al. [24] proposed that during the DS process, there is a definite positional relationship between the γ phase and α phase, so the orientation of the high-temperature α phase must be controlled. They raised four conditions for qualified α seed crystals: (i) the primary phase is α phase; (ii) when heating to α → α_2_/γ transition point, the lamellar structure is stable and α phase simply transforms to α_2_ phase; (iii) during heating, α phase is thermodynamically stable and the volume fraction of α phase increases by thickening of α lamellae rather than new nucleation; (iv) the mentioned process is reversible and the original orientation of lamellar structure can be retained during cooling. Among the alloys that meet the four conditions, Ti-43Al-3Si has been confirmed as a widely applicable seed crystal. Generally, after cutting the Ti-43Al-3Si seed ingot, its lamellar structure is perpendicular to the axial direction and needs to be rotated 90° and bonded to the bottom of the master alloy. Kim et al. [29] improved the efficiency of this method by pouring the melt into a metal mold. Due to the chill effect, α grains grow along the direction <0001>, perpendicular to the inner wall of the mold and parallel to the axial direction. According to the Blackburn relationship, after the seeding process, the lamellae will be parallel to the axial direction.

In recent years, novel research on traditional α seeds has included Du et al. [30,31,32] using Ti-43Al-3Si seed to prepare large-size Ti-47Al samples. They fixed the master alloy rod on the feeding mechanism and the seed rod with the same diameter on the pulling mechanism melted the upper part of the seed crystal and the lower part of the master alloy by electromagnetic induction heating, and then the metal formed a 10~20 mm long melting zone under the electromagnetic restraint. In the initial stage of seeding, a large number of initial α grains were nucleated on the seed and grew along the non-preferred direction <$11\bar{2}0$>. After the competitive growth of α grains in the transition zone, the main part of the rod was only composed of one grain. The maximum width is 16 mm, and the lamellar structure is parallel to the axial direction.

Ti-43Al-3Si is able to seed certain high-Nb-TiAl alloys. According to Yue et al. [4], the seed crystal with the same diameter as the parent alloy was cut and placed beneath the parent alloy Ti–46Al–5Nb-0.18C-0.3Si. After DS, it could be found that the lamellar structure of the seed crystal was coarsened during long-term annealing, but the lamellar orientation was still parallel to the crystal’s growth direction. Although a large number of fine-grained Ti_5_Si_3_ phases were distributed in the seed region, they did not affect the lamellar orientation. During the heating and remelting processes, the lamellar structure became extraordinarily stable due to the presence of a large number of Ti_5_Si_3_. In the transition zone, coarse α dendrites grew along the non-preferred orientation, and Ti_5_Si_3_ almost disappeared in this zone, then completely disappeared in the DS region. This proved that Ti-43Al-3Si may have limited influence on the mechanical properties of the main part of the sample.

The disadvantages of the traditional α seed crystal method mainly lie in the demanding kinetic conditions and long transition zone. Other novel seed methods aim to solve both problems.

### 3.2. Self-Seeding Method

To reduce the negative influence of an overlong transition zone, Fan et al. [17] developed a kind of in-situ self-seeding method. Essentially, it can be seen as an α seed crystal method, but the seed crystal has the same composition as the master alloy. The sample can be directly cut from the parent alloy ingot for conducting DS without the preparation of seeds. The lamellar orientation of the unmelted zone is parallel to the growth direction, while the alloy solidifies in the α phase and can grow in non-preferred orientations under appropriate solidification parameters.

**Figure 2:**
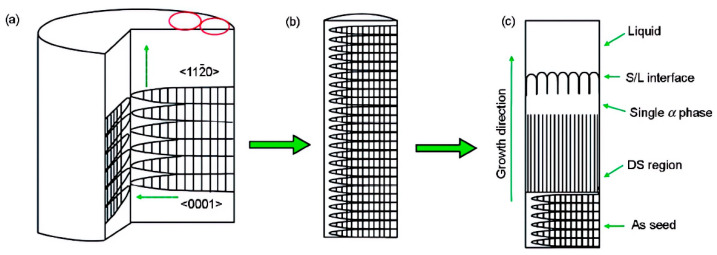
Schematic diagram of preparing specimens for lamellar orientation control by self-seeding method (SST) [33]. (**a**) structures of master ingot, (**b**) structures of seeding specimen, (**c**) solidification processing of SST.

For a specific composition Ti-46Al-0.5W-0.5Si, a cut sample was directly used for DS, and the bottom of the sample acted as a seed; the schematic is shown in Figure 2 [33]. The growth principle of the seed crystal part is similar to that proposed by Kim et al. [34]. Since the seed and the master alloy had the same composition, the length of the transition zone decreased significantly. Although the lamellar orientation in the area of the seed crystal was parallel to the growth direction, with the change of solidification parameters, the angle between the lamellar orientation and the growth direction in the DS region may change in the range of 0°~45°, considering that the growth of grains was affected by both heat flow and interfacial tension, causing the growth direction of α phase to be between the heat flow direction and the preferred growth direction.

Fan et al. [16] also studied the preparation of directionally solidified Ti-49Al alloy by self-seeding. Other alloys that can adopt self-seeding include Ti-50Al-4Nb [18] since the Al-equivalent of composition is similar to Ti-49Al. During the seeding process, it was found that the primary dendrite growth direction of non-preferred grains always deviated from the axial direction at the solid-liquid interface. This may be due to the comprehensive effects of interface energy anisotropy and heat flow direction. In the subsequent growth process, grains with different orientations competed for growth. At low growth rates, utilizing the growth advantages of non-preferred crystals could obtain nearly parallel layered structures. However, at high growth rates, some grains nucleated at the inner wall of the mold and grew poorly oriented grains, which hindered the growth of grains with parallel orientation.

### 3.3. Double DS Self-Seeding Method

The composition of the novel high-Nb-TiAl alloy is very different from that of traditional α seeds, resulting in an excessively long zone and non-negligible performance degradation. Ding et al. [35] demonstrated the double DS self-seeding method, which includes two DS processes. First, determine the solidification parameters and conduct the first DS process of a sample rod so that the primary phase is β, the solidification undergoes a peritectic process. Subsequently, the sample is rotated 180° along the axial direction to conduct the second DS process with exactly the same solidification parameters.

**Figure 3:**
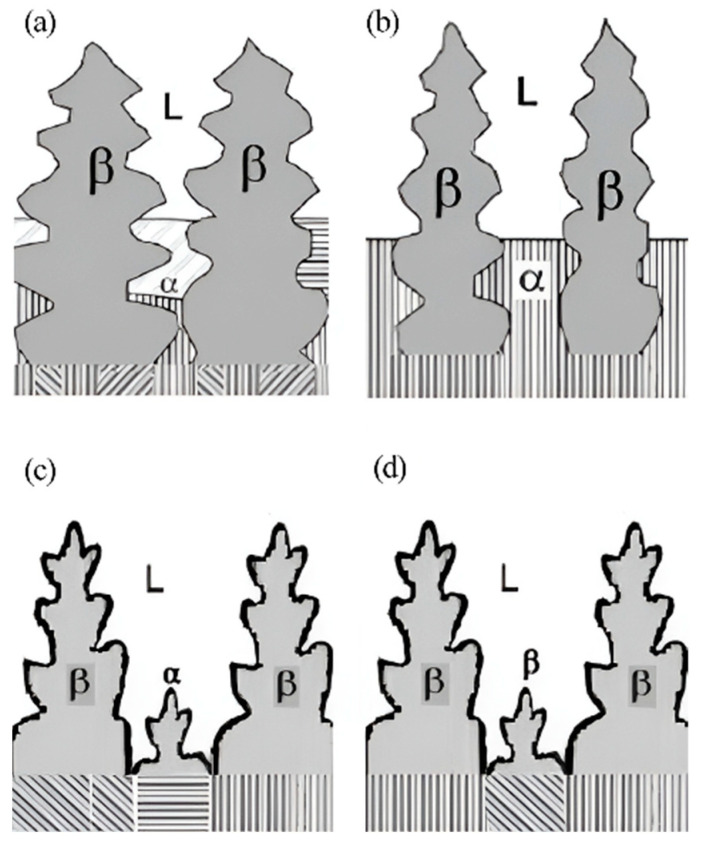
Schematic growth morphologies of TiAl alloys during the second DS, showing the growth morphologies with narrow (**a**), appropriate (**b**), and wide (**c**) interdendritic spacing, and the one (**d**) in the double DS under the different DS conditions. Reprinted with permission from Ref. [36]. 2011, ELSEVIER.

The method was used to prepare a directionally solidified Ti-46Al-5Nb alloy [36]. It was found that the lamellar orientation of some dendrites in the initial growth zone of secondary DS alloy was the same as that in the annealing zone at the withdrawing rate of 30 μm/s, and the angle between lamellar orientation and growth direction was 30°, but in the DS region, the lamellar orientation and growth direction were parallel. The lamellar orientation can be controlled parallel to the axial direction by the complete peritectic transformation L + β → α in dendrites, ensuring the consistency of the high-temperature α phase. The transition was carried out in the dendrite and interdendritic regions as the interdendritic liquid entered the nuclei of β dendrites. The interdendritic spacing is an important parameter in controlling peritectic phase transformation, and the schematic is shown in Figure 3 [36]. If the interdendritic spacing is narrow, the peritectic transformation in the β-dendrite region is incomplete after depletion of the liquid phase. Conversely, if the interdendritic spacing is wide, some α phase may precipitate directly from the liquid phase, resulting in a lamellar structure parallel to the radial direction. Another situation is when single β solidification happens, causing 12 variants, making it difficult to acquire consistent lamellar orientation.

Peritectic reactions can be carried out at relatively low G/v values; hence, the double DS method can be applied to a large range of withdrawing rates, which actually reduces the requirements for DS equipment [37].

### 3.4. Quasi-Seeding Method

In order to solve the limitation of traditional α seed crystals, Du et al. [15] proposed to develop a class of quasi-seed crystals that are different from traditional α seed crystals in that they cannot thermodynamically meet the requirements of α seed crystals but can meet the seeding requirements by controlling kinetic conditions. The primary phase of a quasi-seed crystal is β, and under certain conditions, the quasi-seed solidifies in the form of a peritectic α phase, and then the lamellar structure becomes parallel to the axial direction of the ingot. This requires special heating procedures that ensure the lamellar structure of the quasi-seed crystals remains stable during heating and remelting.

The solidification characteristics of Ti-48Al-2Nb-2Cr and Ti-48Al-6Nb-1Cr alloys are very similar to those of commonly applied Ti-47Al alloys and are suitable as quasi-seed crystals. The characteristics of these two alloys include that the primary phase is β, the solidification mode is hyper-peritectic, and peritectic α solidification is easy to achieve. In addition, the alloys are sensitive to the heat treatment, after which the lamellar orientation is changeable so as to actualize different seeding processes.

The preparation method of the Ti-48Al-2Nb-2Cr quasi-seed crystal was the same as that of the Ti-43Al-3Si seed crystal, mentioned in Section 3.2. The melt was poured into the mold at room temperature, forming a peritectic α phase that nucleated at the inner wall of the mold and grew along the temperature gradient. After natural cooling, the quasi-seeded lamellar structure was parallel to the axial direction. According to the phase selection diagram and the numerical simulation of the solidification process of the seed ingot, it could be found that the range of G/v value is located in the α cell/dendrite solidification area, which proved that the primary phase was α. During heating and remelting, when the heating rate of the stage from 900 °C to 1450 °C was above 61 °C/min, a small amount of precipitation may lead to the generation of γ grains but does not affect the overall stability of lamellar orientation. Subsequently, at a high cooling rate, the lamellae were well preserved.

**Figure 4:**
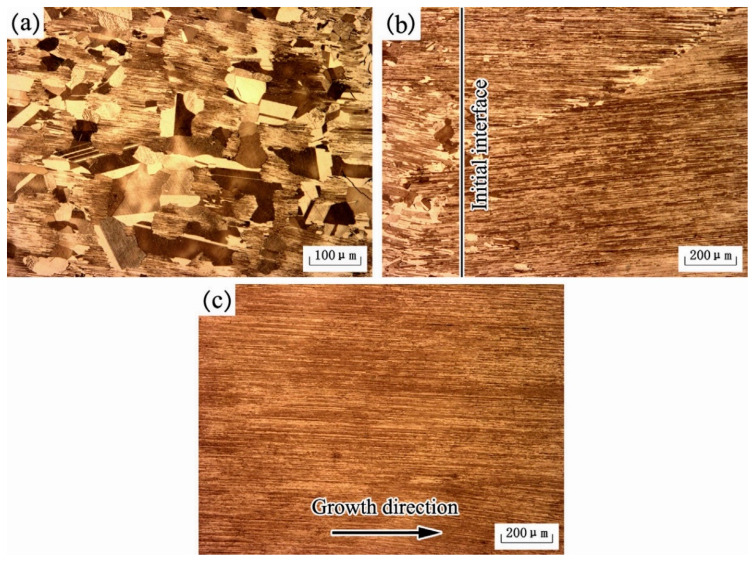
Microstructures of annealing region (**a**), initial interface (**b**), and DS region (**c**) of the electromagnetic confinement directional solidification (EMCDS) sample at the growth velocity of 10 μm/s. Reprinted with permission from Ref. [14]. 2015, ELSEVIER.

Figure 4 [14] describes the microstructure of the alloy at different locations after DS at a withdrawing rate of 10 μm/s, using Ti-48Al-2Nb-2Cr alloy as the seed crystal. The annealing area exhibited a typical duplex microstructure, where high-temperature α grains were transformed from α/γ lamellae during rapid heating well-aligned columnar grains with ideal lamellar microstructure were obtained under the seeding of these α grains. Competitive growth was observed between adjacent columnar grains, eventually forming the stable lamellar structure shown in Figure 4c. The nucleation and growth of γ grains are the most critical factors affecting the stability of the lamellar microstructure during heat treatment. If the nucleation and growth of the γ phase are restricted, high-temperature α grains with poor orientation originating from the γ phase will not be generated in the DS region, and the lamellar microstructure may remain unchanged.

For another example, the lamellar structure of Ti-48Al-6Nb-1Cr remained stable during rapid heating and remelting; in this process, the γ phase did not grow up, despite the fact that a large number of fine equiaxial γ grains appeared at the initial solidification interface. Unsurprisingly, these grains did not affect the seeding process, and the lamellar structure in the final DS region grew continuously and had a fine orientation.

### 3.5. Pure Metal Seeding Method

The principle of using pure metal seeding is that the pure metal can act as a component in the master alloy and form the seed crystal region through the solute exchange; therefore, the seeding progress effectively reduces the length of the transition zone and the influence of the mushy zone. Liu et al. [9,38] systematically elaborated on this method and reported pure Ti or Nb as seed crystals.

**Figure 5:**
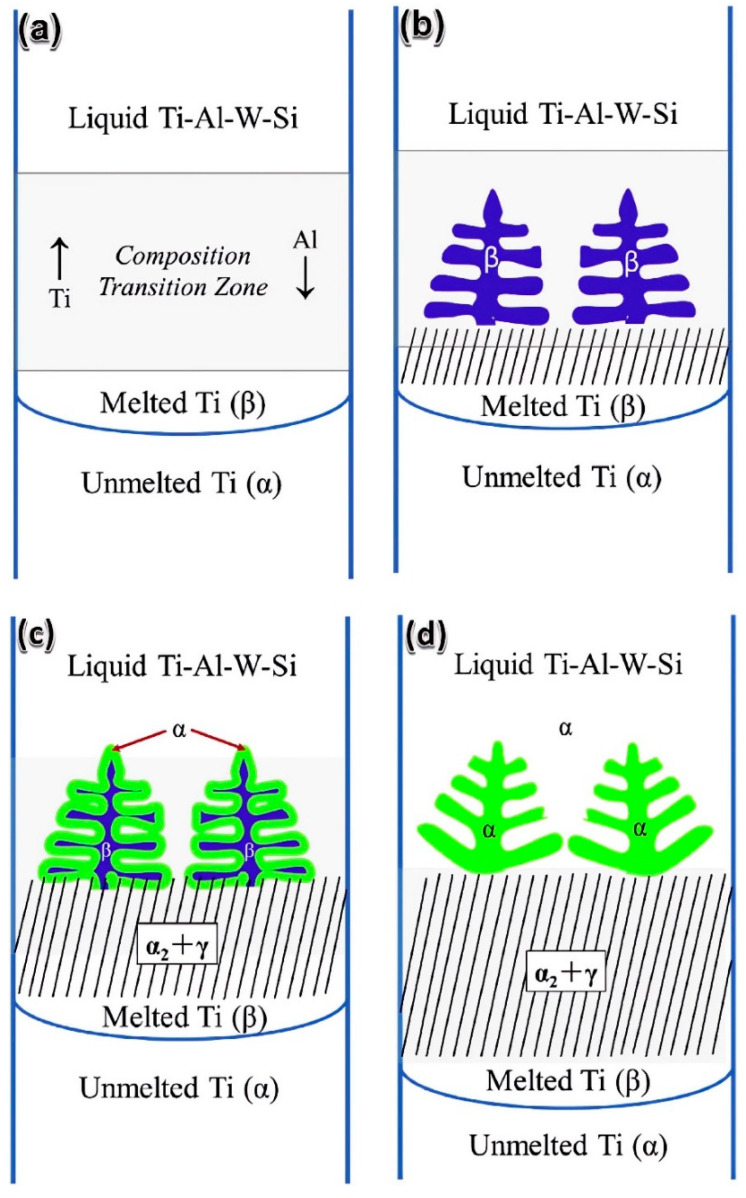
Schematic illustration of the β seeding technique in directionally solidified Ti-47Al-0.5W-0.5Si: (**a**) the form of the composition transition zone; (**b**) the initial directional solidification stage in the composition transition zone; (**c**) the primary β dendrite is transformed into α grain; and (**d**) the growth of α phase in steady-state growth region. Reprinted with permission from Ref. [38]. 2016, ELSEVIER.

Pure Ti was used as a kind of seed crystal in the Ti-47Al-0.5W-0.5Si alloy and the Ti-47Al binary alloy. By connecting the pure Ti seed below the master alloy and ensuring that the bonding interface is lower than the initial DS interface, the sample can be completely melted during the process of heating and thermal treatment. Solute exchange and diffusion occur between the seed and the master alloy. At the beginning of the DS process, the leading β phase grew in preferred orientation <001>. As the Al content rose, leading β dendrites transformed into the α phase through peritectic transformation, and the leading α phase was nucleated on the basis of the α phase that underwent the peritectic transformation and inherited its lamellar orientation, as shown in Figure 5. There were angles of 10–20° between the lamellar and the growth direction, which was due to the certain deviation between the leading phase’s preferred direction and the growth direction.

Since Nb and β-Ti both belong to body-centered cubic structure, the low mismatch degree between Nb and β-Ti causes a completely coherent interface; therefore, β-Ti has a very strong ability for heterogeneous nucleation on the Nb-based interface. This makes it possible for pure Nb to serve as a seed crystal for Ti-47Al-1.0W-0.5Si and similar alloys. Massive Nb addition improved β → α transformation temperature, making the β phase difficult to transform to α phase during the cooling process. A large number of B2 phases were generated at a position close to the crystallization interface. With the further increase in distance, the B2 phase disappeared and a lamellar structure (α_2_ + γ) appeared, in which only Ti_5_Si_3_ existed. Several grains were formed in front of the crystal interface; the angles between the lamellar orientation and the growth direction of each grain were within 45°.

## 4. Controlling Solidification Parameters

As mentioned previously, the lamellar structure is obtained from the α parent phase, which is affected by the solidification process and solid-state phase transition. Controlling solidification parameters involves determining the temperature gradient and the growth rate of the sample to achieve a specific solidification mode, resulting in lamellae approximately parallel to the growth direction. The determination of these parameters is based on the TiAl alloy phase selection diagram and experiments.

During directional solidification, the nucleation process can be explained by the constitutional supercooling theory. As a result of the generally fixed temperature gradient, the undercooling degree of components can be controlled by simply controlling growth rates or withdrawing rates in practical operation. It is crucial to ensure that the undercooling degree of the solidification interface is lower than the nucleation undercooling degree of the primary β phase. Otherwise, new grains will form in front of the solidification interface, and the growth direction will not be influenced by the aligned lamellae.

Chen et al. [11] proposed that the nucleation barrier in the 45° direction is higher than that in the 0° direction; that is, at the same degree of undercooling, there exists a critical velocity named v_c_. When the pulling velocity is higher than v_c_, the angle between the lamellar and growth directions is 45°, or the angle is 0°. This phenomenon can be described by the change in the free energy of the entire system.

Further, Zheng et al. [39] described the change of Gibbs free energy (∆G) when α phase nucleates in β matrix:

∆G = −V∆G_v_ + Aγ + V∆G_s_

**Figure 6:**
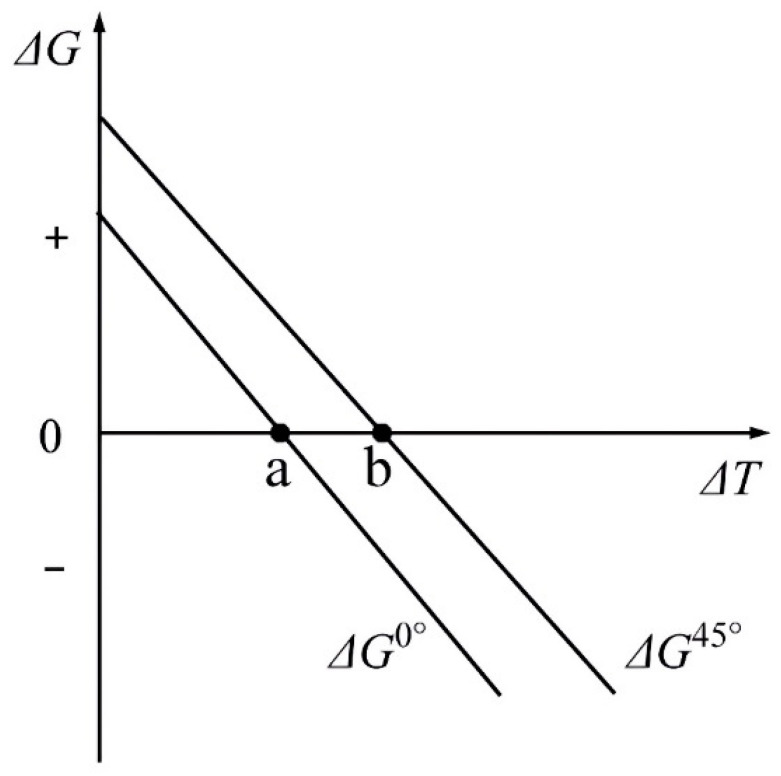
Variation of the free energy change with nucleation undercooling for 0° or 45° oriented growing α phase. Reprinted with permission from Ref. [39]. 2022, MDPI AG.

These results indicate that the Gibbs free energy changes consist of the decrease in volume free energy (−V∆G_v_), the increase of β/α interfacial free energy (Aγ), and the increase of misfit free energy (V∆G_s_). The decrease in volume-free energy is the main driving force of nucleation, but the change in volume-free energy and mismatch-free energy is independent of the crystallographic orientation of α and β. The directional phase transition is considered to be carried out on a two-dimensional plane, where the driving force of the phase transition is parallel to the axial direction, and the different unit area interfacial energy γ perpendicular to the driving force direction caused by different β/α interfaces has an important effect on the lamellar direction. Since the interfacial energy of ($00\bar{1}$)_β_//($11\bar{2}0$)_α_ is lower than that of ($00\bar{1}$)_β_//($10\bar{1}2$)_α_, the 0° lamellar structure is easier to form than the 45° lamellar structure under general circumstances, but the 45° lamellar structure forms easier when the undercooling degree is sufficient. The schematic is illustrated in Figure 6.

Similar conclusions were obtained by Fan et al. [40,41,42,43] on the solidification parameters of Ti-49Al. The degree of undercooling must be lower than the degree of nucleation undercooling in the primary β phase. When the growth rate was taken as a variable, the degree of interfacial undercooling decreased first and then increased. The growth rate below the undercooling degree of the β phase ranged from v_1_ to v_2_, which could ensure the subsequent continuous growth of the α phase. As the temperature gradient increased, the range of growth rates expanded. When the temperature gradient was taken as a variable, the trend was similar. A temperature gradient ranging from G_1_ to G_2_ allowed continuous growth of the α phase, and the value of G_1_ ascends with an increasing growth rate. A higher G ensured continuous growth at a higher v. When G was 12.1 K/mm and v was 20 μm/s, the α phase grew along <$11\bar{2}0$>_α_ direction, leading the lamellae orientation close to 0°.

Liu et al. [44] believed that the growth rate of Ti-47Al could be directly controlled without using seed crystals. When the growth rate was 10 μm/s, the angle between lamellar orientation and growth direction was 45°, while the angle was 0° at a growth rate of 3 or 20 μm/s. For all three growth rates, the primary phase was α and remained unchanged, inferring that the initial DS interface affected the lamellar orientation.

When the growth rate varies greatly, it is easy to undergo a change in solidification mode on most occasions. Jiang et al. [45] found that for Ti-44Al-9Nb-1Cr-0.2W-0.2Y alloy, within the range of growth rate of 10~20 μm/s, as the growth rate increased, the angles between the columnar grain orientation and the ingot axial direction gradually decreased, and the preferred grain orientation gradually changed from the heat flow direction to the pulling direction. Moreover, the difference in growth rate caused changes in the primary solidification phase. Initially, it solidified as a single β phase, but when the growth rate reached 20 μm/s, the lamellar orientation included cases where it formed hypo-peritectic solidification with angles of 45° or 90° to the growth direction, indicating that single-α solidification occurred. Wang et al. [46] found that for Ti-47Al-6Nb-0.1C alloy, at growth rates of 5 μm/s or 8.33 μm/s, the lamellae with small angles to the growth direction accounted for the major proportion because of primary β solidification. The angles increased with the increase in growth rates. When the growth rate increased to 16.67 μm/s, lamellae with a near 90° angle accounted for the major proportion, which is related to primary α solidification.

**Figure 7:**
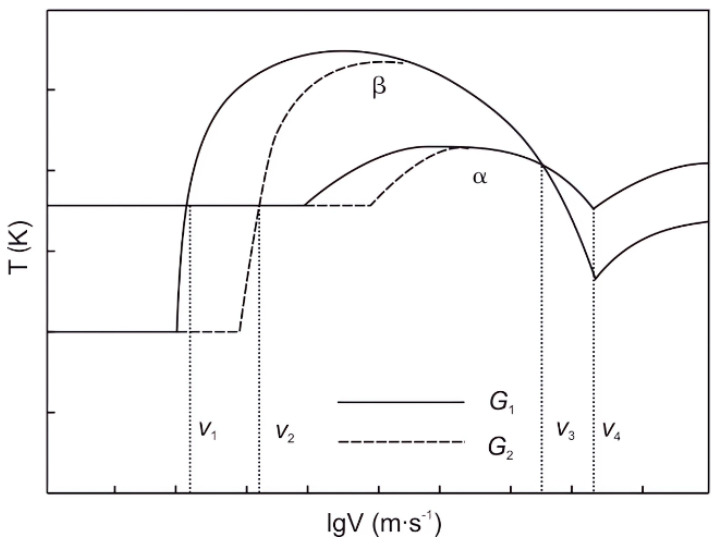
Schematic of interfacial temperature response function with growth rate and temperature gradient. Reprinted with permission from Ref. [47]. 2016, Springer Nature.

The research by Luo et al. [47] on the Ti-45Al-5Nb alloy revealed similar patterns. Within a growth rate range of 5~20 μm/s and a temperature gradient range of 15~20 K/mm, the primary solidification phase was β, and the alloy underwent the following β → α and α → α_2_ + γ transformations, resulting in lamellae at angles of 0° or 45° to the growth direction. At a temperature gradient of 20 K/mm and a growth rate higher than 20 μm/s, the primary solidification phase changed from β to α, and the alloy underwent single-phase α solidification, resulting in lamellae at an angle of 90° to the growth direction. The determination of the primary phase can generally be based on the theory of the interface temperature response function, as shown in Figure 7.

Since it is difficult for most alloy compositions to obtain proper lamellar direction only by controlling solidification parameters. In most cases, seed crystals are used jointly.

## 5. Novel Ways for Controlling Lamellar Orientation

Thermal stabilization (TS) or heat treatment (HT) is also used to optimize lamellar orientation. Generally, the heat preservation before DS is called TS, and the heating operation on the sample after DS is called HT. TS ensures a higher temperature gradient and promotes the stable formation of the transition zone, while HT effectively optimizes the orientation and uniformity of the lamellae.

Ding et al. [48] first studied the post-solidification HT of Ti-45Al-8.5Nb. Two HT procedures, HT1 (1250 °C for 24 h + 900 °C for 30 min + air cooling), and HT2 (1400 °C for 12 h + 900 °C for 30 min + air cooling), were selected. After HT1, lamellar orientation did not change significantly, but after HT2, lamellar orientation became parallel to the growth direction. This is due to the fact that the maximum temperature of HT1 was too low to reach the α transition point, so the lamellae are not decomposed. The HT2 temperature promoted the fusion of the high-temperature α phase, and then the macrostructure was close to a single crystal.

Zhang et al. [49] performed thermal stabilization on Ti-46Al-5Nb alloy before DS and proved that proper TS is necessary to generate L + β + α three-phase zone in the mushy zone, which benefits the control of the microstructure in the DS zone of peritectic TiAl alloy. When the TS time was 30 min, a three-phase zone appeared in the mushy zone. As the TS time increased to 60 min, the liquid in the mushy region disappeared, and columnar β and α grains could be observed in the mushy zone, indicating that the near-steady state had been reached during the TS period. After remelting, the crystal grew during DS, relying on the high-temperature interface in the mushy region, which inherited the crystal orientation formed during TS. Specifically, the α phase recovers on the basis of the β phase orientation through L + β → α peritectic transformation, and a parent α grain usually forms only one lamellar colony during cooling. At the same time, the TS treatment can make the β dendrites grow evenly and make the same peritectic transformation occur in different β dendrites, so as to obtain the lamellar parallel to the axial columnar crystals.

**Figure 8:**
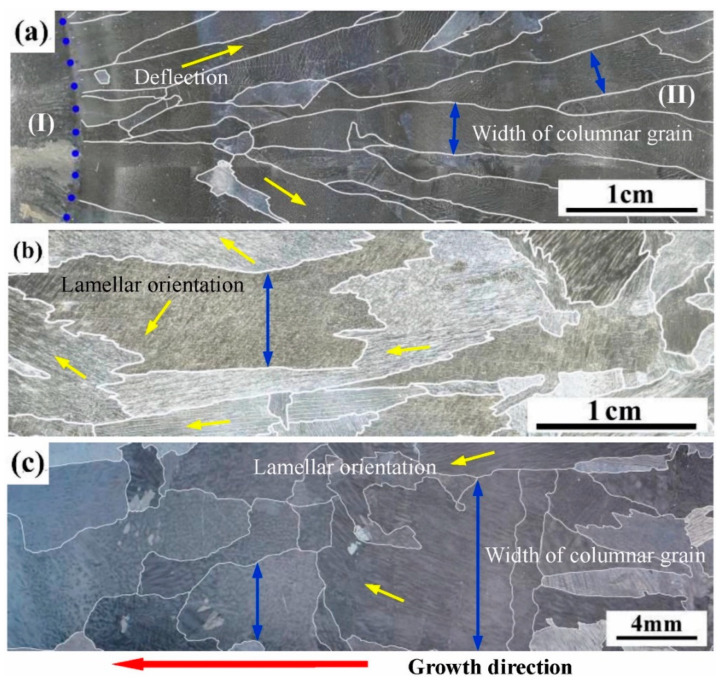
Macrostructure of Ti-44Al-6Nb-1Cr alloys. (**a**) Directionally solidified alloy; (**b**) DHT-1 alloy; and (**c**) DHT-4 alloy. Reprinted with permission from Ref. [50]. 2021, ELSEVIER.

Directional heat treatment (DHT) requires the sample to be pulled into the cooling medium at a certain rate during the HT process. After DS, Chen et al. [50] conducted DHT for Ti-44Al-6Nb-1Cr alloy at a temperature of 1750 K and a pulling rate of 4.17 μm/s. The temperature was chosen based on the findings of Liu et al. [51], who demonstrated that for TiAl alloys with original β solidification characteristics, the heat treatment temperature should lie within the β phase region. After a single DHT, a complete single β transition occurred in the effectively heat-treated grains, followed by α → α_2_ + γ processes. Therefore, the angle between the orientation of most lamellar structures and the growth direction of columnar grains was observed to be 0° or 45°. With the increase in the number of DHT cycles, the lamellar colonies grew, and the columnar grain size obviously increased, as shown in Figure 8. Simultaneously, the improvement in the orientation of the lamellar clusters was significant. After four DHT cycles, they observed that all the lamellar clusters derived from a single columnar grain exhibited the same orientation.

Chen et al. [6] also conducted DHT on Ti-44Al-6Nb-1Cr-2V samples at a temperature of 1730 K and a growth rate of 4.17 μm/s. Before heat treatment, it was found that the samples had obtained α_2_/γ full lamellar structure through a typical peritectic transformation. After a single DHT, the average angle between the alloy grains and the axial direction became significantly smaller, while the grains near the surface showed a slighter deflection. Considering that the angle between the arrangement direction of lamellar phases and the axial direction of columnar grains did not change, apparently from a macroscopic point of view, DHT slightly optimized the lamellar orientation of the alloy.

Li et al. [52] investigated lamellar orientation control of β-solidified Ti-43.5Al-4Nb-1Mo-0.1B alloy using a high withdrawing rate combined with thermal stabilization. The thermal stabilization process involved holding the sample at 1973 K for an hour to establish a stable liquid-solid phase and β/α phase interface, as well as creating a relatively high temperature gradient. Additionally, the (111)_β_ plane facing the melt exhibited the lowest loss rate of interface atoms during the melting process, while other β grains on different crystal planes were more prone to lose. The thermal stabilization resulted in the expansion and fusion of β grains, and accordingly, β grains with suitable orientation contributed to the subsequent generation of single crystals. A high withdrawing rate of 100 μm/s could also ensure the grain selection of the α phase and lead to complete peritectic transformation. In the competition growth process of grains, α grains with lamellae parallel to the axial direction would dominate and ultimately form a near PST crystal.

## 6. Conclusions and Prospects of Lamellar Orientation Control

(1) There are variations in the application scenarios for seed techniques and methods for controlling solidification parameters. The traditional α seed crystal method and the pure metal seeding method have certain requirements for the composition of the master alloy and the planar growth of α phase interface. The double DS self-seeding method and the quasi-seed method require the occurrence of peritectic transformation during the solidification process, thus obtaining a lamellar an orientation close to 0°. Controlling solidification parameters demands the growth of the entire β phase during the solidification process, and by precisely adjusting the withdrawing rate, the occurrence of complete peritectic transformation is controlled, and lamellar structure parallel to the growth direction can be obtained.

(2) Further research on controlling lamellar orientation should focus on simplifying the preparation of seed crystals, determining solidification parameters, and improving the heat treatment process. The double DS self-seeding method allows for obtaining the seed crystal directly by cutting a sample after single DS, eliminating the need for casting a seed crystal ingot. Precise control of the growth rate can effectively promote the growth of directional columnar crystals or single crystals, and the temperature gradient can be adjusted by controlling the output power of the equipment using a combination of machine learning algorithms. Before the withdrawal process, the sample should undergo thermal stabilization treatment, followed by heat treatment after DS. To optimize the lamellar orientation, the heat treatment temperature should be above the α transition point. The combination of the seed crystal method and controlling solidification parameters is particularly practical for controlling the lamellar orientation of large samples.

## Data Availability

No new data were created.
